# Evaluation of lung flute in sputum samples for molecular analysis of lung cancer

**DOI:** 10.1186/2001-1326-2-15

**Published:** 2013-09-22

**Authors:** Nigar Anjuman, Ning Li, Maria Guarnera, Sanford A Stass, Feng Jiang

**Affiliations:** 1 Departments of Pathology, University of Maryland School of Medicine, 10 South Pine Street, MSTF 7th floor, Baltimore, MD 21201-1192, USA

**Keywords:** Lung flute, Sputum, microRNAs, Lung cancer, Diagnosis

## Abstract

**Background:**

Molecular analysis of sputum provides a promising approach for lung cancer diagnosis, yet is limited by the difficulty in collecting the specimens from individuals who can’t spontaneously expectorate sputum. Lung Flute is a small self-powered audio device that can induce sputum by generating sound waves and vibrating in the airways of the lungs. Here we propose to evaluate the usefulness of Lung Flute for sputum sampling to assist diagnosis of lung cancer.

**Methods:**

Forty-three stage I lung cancer patients and 47 cancer-free individuals who couldn’t spontaneously cough sputum were instructed to use Lung Flute for sputum sampling. Expressions of two microRNAs, miRs-31 and 210, were determined in the specimens by qRT-PCR. The results were compared with sputum cytology.

**Results:**

Sputum was easily collected from 39 of 43 (90.7%) lung cancer patients and 42 of 47 (89.4%) controls with volume ranges from 1 to 5 ml (median, 2.6 ml). The specimens had less than 4% oral squamous cells, indicating that sputum was obtained from low respiratory tract. Expressions of miRs-31 and 210 in sputum were considerably higher in cancer patients than cancer-free individuals (8.990 *vs.* 4.514; 0.6847 *vs*. 0.3317; all P <0.001). Combined use of the two miRNAs produced a significantly higher sensitivity (61.5% *vs*. 35.9%, P = 0.002) and a slightly lower specificity (90.5% *vs*. 95.2%, p = 0.03) compared with cytology for lung cancer diagnosis.

**Conclusion:**

Lung Flute could potentially be useful in convenient and efficient collection of sputum for molecular diagnosis of lung cancer.

## Background

Lung cancer is the leading cause of cancer death in men and women. Non-small cell lung cancer (NSCLC) is the most common type of lung cancer, mainly consisting of adenocarcinomas (AC) and squamous cell carcinomas (SCC). The early detection of NSCLC followed by appropriate treatments will reduce the mortality [1]. Low-dose CT scan to screen for lung cancer in heavy smokers can reduce the death rate from the malignancy by 20% over chest X-rays [2]. However, CT screening has only 61% specificity over the 3-year screening period [2]. The low specificity often results in anxiety, unnecessary biopsies, and surgeries that carry their own risks to many smokers who have benign diseases. Noninvasive approaches that can help diagnose early stage lung cancer are urgently desired.

Sputum is a noninvasively accessible body fluid that contains exfoliated bronchial epithelial cells [3]. Sputum cytology can identify morphological abnormalities of bronchial epitheliums. However, it has a poor sensitivity [4]. Molecular study of sputum could detect the cells containing lung tumor-associated molecular aberrations that occur in microscopically normal appearing epitheliums, thus providing a promising approach for diagnosis of lung cancer [5-7]. For instance, p16 hypermethylation was found in sputum collected from patients with lung cancer 5–35 months before cytological and clinical diagnoses [8]. Analysis of numerical chromosome changes could predict lung cancer 18 months before clinical diagnosis [9]. Quantification of microRNA (miRNA) expression in clinical specimens could offer a novel approach for cancer screening and early detection [10-13]. Previously, we found that microRNAs (miRNAs) were stably present in sputum and reliably measurable by using quantitative reverse transcriptase PCR (qRT-PCR) assay [14-16]. We further analyzed 12 lung cancer-associated miRNAs in sputum and compared with CT scan for diagnosis of lung cancer in 66 lung cancer patients and 68 cancer-free control subjects. We found that two miRNAs (miR-31 and miR-210) when used in combination produced 65.2% sensitivity and 89.7% specificity. Notably, integrating the sputum miRNA biomarkers and CT yielded a significant higher specificity than CT used alone (91.2% *vs.* 83.8%, P < 0.05). Therefore, the assessment of miRNAs in sputum might be useful to augment CT, particularly increase its specificity, for the early detection of lung cancer.

However, very often some subjects are not able to spontaneously expectorate sputum. Alternately, sputum induction with inhalation of hypertonic saline mist has been used to obtain adequate sputum samples [17,18]. Yet the approach is limited by laborious and time consuming, need for specialized equipment, and the risk of bronchospasm and respiratory inflammation. Therefore, there is an unmet need to develop a safe, simple, and efficient means for efficiently and safely collecting sputum.

Lung Flute is a small self-powered audio device consisting of a mouthpiece and a reed inside a 36.8-cm rectangular plastic tube [19]. Lung Flute can create sound with a frequency of 18–22 Hz [19]. This sound wave, when generated at the mouth by mild exhalation, could vibrate in the airways and cilia. The produced sound resonates with the natural frequency and augments mucus clearing system in the airways, and hence makes mucus secretions thinner and more easily expelled by coughing. Previously, Fujita et al. found that Lung Flute could efficiently induce sputum from tracheobronchial and low respiratory track [19]. Furthermore, the sputum samples collected by using this technique had no significant differences in biological markers or cell counts compared with those induced using hypertonic saline in patients with chronic bronchitis. In addition, Fujita et al. [19] suggested that although Lung Flute depends on patient effort, it was noninvasive and easy to use. The device did not require special equipment, and the patient didn’t have an empty stomach before using it. Patients could easily carry the device and use it at home repetitively [19]. Moreover, the use of Lung Flute for sputum sampling could help rapidly diagnose pulmonary tuberculosis [19]. However, there has no report of investigating its utility in collecting sputum for diagnosis of lung cancer.

The goals of the study were to evaluate whether 1) the use of Lung Flute could safely and effectively collect sputum from lung cancer patients and cancer-free individuals who were not able to spontaneously produce sputum, and whether 2) analysis of miRNAs in the sputum samples might facilitate diagnosis of lung cancer.

## Methods

### Patients and sample collection and preparation

Eligible lung cancer patients were stage I NSCLC patients before the patients receiving surgical treatment, preoperative adjuvant chemotherapy and radiotherapy. The research protocol for the study was approved by Institutional Review Board of University of Maryland Medical Center. Inclusion criteria for cancer-free controls were individuals who had no a history of cancer in the last 3 years at the time of enrolment. Furthermore, enrollment was restricted to lung cancer cases and controls who were not able to expectorate spontaneous sputum. Exclusion criteria for both cancer and cancer-free patients were 1) exacerbation of chronic obstructive pulmonary disease (COPD) or hospitalization for COPD within 8 weeks prior to enrollment, 2) predominant asthma and bronchiectasis by clinical assessment, 3) history of cough syncope, 4) pregnant or nursing women, and 5) inability to comply with study procedures. Written informed consent for participation was obtained through an institutional review board-approved protocol. Final diagnosis for lung cancer was made with histopathologic examinations of specimens obtained by CT-guided transthoracic needle biopsy, transbronchial biopsy, videotape-assisted thoracoscopic surgery, or surgical resection. The surgical pathologic staging was determined according to the TNM classification of the International Union Against Cancer with the American Joint Committee on Cancer and the International Staging System for Lung Cancer. Histopathological classification was determined according to the World Health Organization classification. The diagnosis of COPD was established according to the standards of the Global Initiative for Chronic Obstructive Lung Disease [20].

Sputum was collected by using Lung Flute (Medical Acoustics, Buffalo, NY) according to the manufacturer’s instruction and the method previously described [19]. Briefly, after blowing nose and rinsing mouth to minimize contamination of squamous cells from postnasal drip and saliva, the participants placed lips around mouthpiece. They blew out fast and hard through Lung Flute. After taking a quick breath, the participants blew out again, and then coughed up sputum into a sterile container. The samples were processed on ice in four volumes of 0.1% dithiothreitol (Sigma-Aldrich Corporation, Saint Louis, MO), and the mixture was vortexed for 15 min. A double volume of a phosphate-buffered saline solution was then added, and the mixture was briefly vortexed. After filtration through two layers of a sterile gauze to remove mucous and debris, sputum was centrifuged for 10 min at 800 g. The cell pellet was resuspended in a phosphate-buffered saline solution. A differential cell count was performed using slides stained with the May-Grünwald-Giemsa method. The sputum specimens were of low respiratory origin as indicated by the presence of less than 4% oral squamous cells. Slides were considered insufficient for diagnosis, if they had zero to three histiocytes, excessive cellular degeneration, or obscuring bacterial and fungal contaminations.

### Cytology

Cytocentrifuge slides were prepared from each sputum sample by using a cytospin machine (Shandon, Inc., Pittsburgh, PA). The slides were then fixed in 95% alcohol for Papanicolaou’s staining [21]. Cytologic diagnosis was performed based on a seven-tiered scoring system as follows: negative, squamous metaplasia, mild dysplasia, moderate dysplasia, severe dysplasia, carcinoma, or insufficient for diagnosis. Positive cytology included severe dysplasia and carcinoma [21,22].

### Isolation of RNA and analysis of miRNAs by qRT-PCR

Total RNA containing small RNA was extracted from sputum as previously described [14]. The purity and concentration of RNA were determined from OD260/280 readings using a dual beam UV spectrophotometer (Eppendorf AG, Hamburg, Germany). RNA integrity was determined by capillary electrophoresis using the RNA 6000 Nano Lab-on-a-Chip kit and the Bioanalyzer 2100 (Agilent Technologies, Santa Clara, CA).

miRs-31 and 210, which were previously identified as potential sputum miRNA biomarkers [14-16], were evaluated in the sputum specimens by using qRT-PCR with Taqman miRNA assays (Applied Biosystems, Foster City, CA) [14-16]. Briefly, RNA was applied for reverse transcription (RT) by using the Applied Biosystems 9700 Thermocycler (Applied Biosystems) with miRNA-specific looped primer and TaqMan® MicroRNA RT Kit (Applied Biosystems). The reaction includes 50 nM stem-loop RT primers, 1× RT buffer, 0.25 mM each of dNTPs, and 3.33 U/μl MultiScribe reverse transcriptase in a total volume of 15 μL. The 20 μl PCR reaction included RT product, 1× TaqMan® Universal PCR Master Mix (Applied Biosystems), and the corresponding primers and Taqman probes for the target genes. The reactions were incubated in a 94-well plate at 95°C for 15 min, followed by 45 cycles of 95°C for 15 s and 60°C for 1 min. Threshold cycle (Ct) values of the miRNAs were normalized in relation to that of U6. All assays were performed in triplicates, and one no-template control and two interplate controls were carried along in each experiment.

### Statistical analysis

Pearson’s correlation coefficient tests with multivariate regression analysis were performed to evaluate the associations between expressions of the miRNAs and clinicopathologic and demographic characteristics. Receiver-operating characteristic (ROC) analysis was undertaken using expression level for each miRNA in lung cancer patients and cancer-free controls [23]. The cut-off value was chosen at the point of highest Youden Index for each miRNA from the ROC in the set of cases and controls. All P values were two sided, and a P value of <0.05 was considered statistically significant.

## Results

### Collecting sputum by using lung flute

Fourty-three lung cancer cases and 47 controls with COPD who were not able to spontaneously cough sputum were recruited in the current study. Sputum was collected by using Lung Flute from 39 of the 43 (90.7%) lung cancer patients and 42 of the 47 (89.4%) cancer-free controls. Characteristics of the 39 lung cancer patients and 42 cancer-free individuals are shown in Table 1. The lung cancer patients are diagnosed with stage I NSCLC. Of the 39 NSCLC patients, 21 are classified with AC and 18 with SCC. The age of the lung cancer cases is 65.6 ± 7.8 year old and 23 (58.9%) are males. The number of smoking pack-years of the lung cancer cases is 48.5 ± 35.3. Of the lung cancer patients, there are 36 (92.3%) smokers with >30 pack-years with COPD, and three non-smokers. The 42 cancer-free subjects include 38 (90.5%) with COPD, one with pneumonia, two with sarcoidosis, and one with inflammatory granuloma. The cancer-free subjects consist of 26 males (60.5%). The age of the control subjects is 62.3 ± 8.4 years old. The number of smoking pack-years of the control subjects is 46.7 ± 32.8. Of the 42 cancer-free subjects, there are 38 smokers with >30 pack-years, and four non-smokers.

**Table 1:** Demographic and clinical data of lung cancer patients and cancer-free controls

	**Lung cancer patients**	**%**	**Cancer-free controls**	**%**
**Parameter**	**Mean**		**Mean**	
Total no.	39		42	
Age, years	65.6		62.3	
	7.8		8.4	
Gender				
Men	23	58.9	26	60.5
Women	16	41.1	16	39.5
Race				
White American	29	74.4	31	73.8
African American	10	25.6	11	26.2
Smoking, pack-years	48.5		46.7	
	35.3		32.8	
Histology				
Adenocarcinoma	21	53.9		
Squamous cell carcinoma	18	46.1		
All are stage I NSCLC				

*Abbreviation: NSCLC* non-small cell lung cancer.

The individuals could expectorate sputum within 20 minutes after using Lung Flute. The median volume of the sputum sample was 2.6 ml, ranging from 1.0 to 5.0 ml. There was not statistical difference of the success rate of the collection and volume of the specimens regarding smoking status, diagnosis of lung cancer, and pulmonary function. There were no statistical differences of differential cell count between the lung cancer patients and cancer-free controls (P > 0.05) (Additional file 1: Table S1). Side effects of using Lung Flute include 8.6% (7 of 81) individuals complained of minor sore throat or dizziness. However, the slight discomforts could be reduced by drinking water and taking several slow breaths.

### Expressions of the miRNAs in lung flute collected-sputum and the diagnostic significance for lung cancer

**Figure 1:**
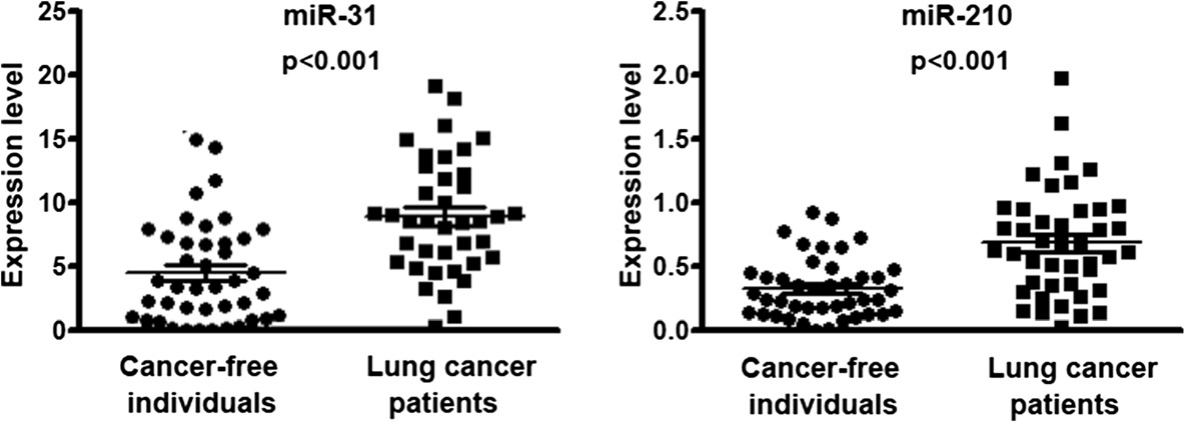
**The expression levels of two miRNAs, miRs-31 and 210, were assessed in sputum of 39 stage I NSCLC patients and 42 cancer-free individuals using qRT-PCR.** U6 small nuclear RNA was used as an internal control.

Expressions of the two miRNAs, miRs-31 and 210, were successfully measured in sputum by using qRT-PCR assay that always yielded a ≤32 Ct value for each miRNA gene. As shown in Figure 1, miR-31 and miR-210 exhibited higher expression levels in sputum of stage I lung cancer patients compared to cancer-free subjects (8.990 *vs.* 4.514; 0.6847 vs. 0.3317, respectively; all P <0.001). Furthermore, the expression levels in sputum were positively associated with smoking history of lung cancer patients (P = 0.032 and 0.026, respectively). However, there was no statistically significant correlation of the changes of the miRNAs with age, ethnicity, and histological type of lung cancer (All p > 0.05).

**Figure 2:**
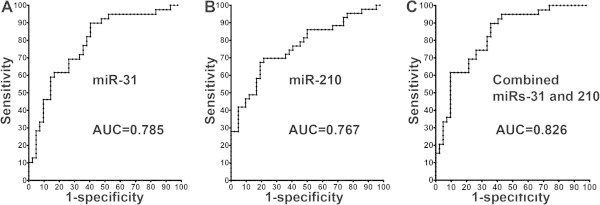
**Receiver-operator characteristic (ROC) curve analysis of expression levels of the two miRNAs (miR-31 and miR-210) in sputum of 39 patients diagnosed with stage I NSCLC and 42 cancer-free individuals.** miR-31 and miR-210 produce 0.785-0.767 AUC values **(A-B)**, being significantly lower than 0.826 AUC created from combined use of two miRNAs **(C)** (P < 0.05).

The two miRNAs exhibited area under the ROC curve (AUC) values of 0.785 and 0.767 in distinguishing lung cancer cases from control subjects, respectively (Table 2) (Figure 2). Interestingly, combined use of the two miRNAs produced 0.826 AUC, offering superior performance compared to any of the two miRNAs used alone (All P < 0.05) (Table 2) (Figure 2). As a result, combined analysis of the two miRNAs generated 61.5% sensitivity and 90.5% specificity for diagnosis of lung cancer.

**Table 2:** The AUCs and corresponding sensitivity and specificity of the two miRNAs that display different expression betwee lung cancer patients and controls

**miRNAs**	**P-value**	**AUC**	**95% confidence interval**	**Std. error**
miR-31	<0.001	0.785	0.6847 to 0.8855	0.05119
miR-210	<0.001	0.767	0.6662 to 0.8676	0.05136
Combined use of miR-31 and 210	<0.001	0.826	0.7366 to 0.9154	0.04559

*Abbreviations: AUC* the area under receiver operating characteristic curve, *CI* confidence interval.

### Comparison of sputum cytology and analysis of miRNAs for lung cancer diagnosis

**Figure 3:**
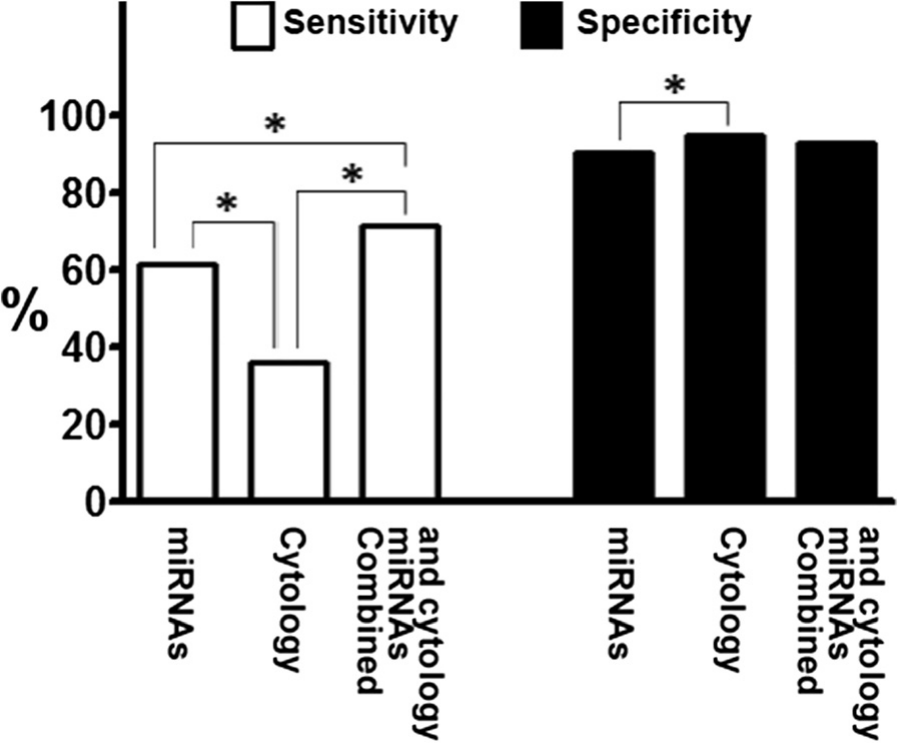
**Comparison of miRNA biomarkers and sputum cytology for lung cancer diagnosis in 39 patients diagnosed with stage I NSCLC and 42 cancer-free individuals.** Analysis of the two miRNAs (miR-31 and miR-210) in sputum produces a higher specificity, a lower specificity compared with sputum cytology. Combined analysis of the miRNAs and cytology presents a higher sensitivity compared with a single approach, maintaining a high specificity at 93.0%. *, statistical significance (P < 0.05).

Cytologic diagnoses of squamous metaplasia and mild, moderate, severe dysplasia, and carcinoma were significantly associated with lung cancer status (Additional file 2: Table S2). For instance, of 42 patients without cancer, 22 (52.4%) were negative by cytology. Conversely, of 39 lung cancer patients, only three (7.7%) were negative by cytology. Furthermore, a diagnosis of severe dysplasia or carcinoma was associated with cancer in 14 lung cancer cases, whereas a diagnosis of severe dysplasia or carcinoma was found in only two patients without lung cancer. If severe dysplasia or carcinoma was considered to be the predictors of the presence of lung cancer, the sensitivity and specificity of the cytological examination of the sputum samples was 35.9% and 95.2%, respectively. When compared with analysis of the miRNAs in the samples, cytology had a considerably lower sensitivity for lung cancer diagnosis (35.9% *vs.* 61.5%, p < 0.01) (Figure 3). However, analysis of the miRNAs displayed a lower specificity compared with sputum cytology (90.5% *vs.* 95.2%, P = 0.003) (Figure 3). Interestingly, integrating the analysis of the miRNAs with cytology offered a higher sensitivity than did any single approach used alone (71.8% vs. 61.5%; 71.8% vs. 35.9%, all P < 0.05), while having 93.0% specificity (Figure 3).

## Discussion

In the present study, we demonstrate that the success rate of sputum sampling is approximately 90%, similar to the reported rate [17] of sputum induction by inhalation of hypertonic saline. The median volume of the sputum samples collected by Lung Flute is 2.6 ml. Sputum is of low respiratory origin as indicated by the presence of less than 4% oral squamous cells. Therefore, the use of Lung Flute can collect sputum with appropriate amount and quality for cytological and molecular studies. Furthermore, the success rate of sputum sampling by using Lung Flute and sample volume don’t display significant difference between smokers and nonsmokers, and are not associated with numbers of pack years as well. The parameters are also not associated with pulmonary function status of the participants. In addition, the small device is user-friendly, because only minor discomforts, such as dizziness and sore throat, are observed in a small percentage of participants. The slight discomforts could easily be remedied. There is no major adverse effect, including bronchoconstriction and respiratory inflammation in the participants when using Lung Flute. Taken together, the findings from our present study reveal that the use of Lung Flute could safely and effectively collect sputum from lung cancer patients and cancer-free individuals who were not able to spontaneously produce sputum.

Our study also shows that the assessment of the miRNAs in Lung Flute-collected sputum produces a higher sensitivity compared with conventional sputum cytology. Furthermore, combined use of the miRNA biomarkers and cytological analysis in the sputum specimens has synergetic efficiency for diagnosis of lung cancer demonstrated by a considerably higher sensitivity compared with each approach used alone. Therefore, the small device could provide a potential tool to facilitate diagnosis of lung cancer through analysis of sputum. The Lung Flute-collected sputum specimens might also have the potential to be used for studying other forms of biomarkers in the early detection of lung cancer. For example, methylations and mutations of lung cancer-related genes could be examined by PCR, or genomic aberrations of lung cancer-related genes by fluorescence *in situ* hybridization in the specimens [8,9,22]. We will undertake a project to compare performance characteristics of the miRNAs to that of the previously developed biomarkers using Lung Flute-obtained sputum specimens. We are carrying out a study in a large population-based case–control study to validate the performance of Lung Flute, compare the efficiency between Lung Flute and sputum induction with inhalation of hypertonic saline mist, and evaluate the contraindication to the Lung Flute.

## Conclusions

Our study indicates that the use of Lung Flute can conveniently and efficiently obtain appropriate sputum from low reparatory track of individuals who are not able to expectorate spontaneous sputum. The application of the small device might overcome a major obstacle of applying cytological and molecular analyses of sputum for lung cancer early detection and screening. However, the diagnostic value using Lung Flute for lung cancer should be validated in independent and prospective case-controlled cohort studies.

## Abbreviations

NSCLC: Non-small cell lung cancer; AC: Adenocarcinomas; SCC: Squamous cell carcinomas; miRNAs: microRNAs; qRT-PCR: quantitative reverse transcriptase PCR; COPD: Chronic obstructive pulmonary disease; CT: Threshold cycle (Ct); ROC: Receiver-operating characteristic; AUC: Area under the ROC curve.

## Competing interests

The authors declare that they have no competing interests.

## Authors’ contributions

NA and MG collected specimens and reviewed clinical information. NL performed molecular and analysis. SS and FJ conceived the study project, organized the whole study process, provided financial support, and finalized the manuscript. All authors read and approved the final manuscript.

## Supplementary Material

Additional file 1: Table S1 Comparison of differential cell counts in sputum samples collected by Lung Flute from cancer patients and cancer-free individuals*. Click here for file

Additional file 2: Table S2 Cytologic diagnosis in lung cancer patients and cancer-free controls. Click here for file
